# Analysis of Prognostic Risk Factors Determining Poor Functional Recovery After Comprehensive Rehabilitation Including Motor-Imagery Brain-Computer Interface Training in Stroke Patients: A Prospective Study

**DOI:** 10.3389/fneur.2021.661816

**Published:** 2021-06-10

**Authors:** Qiong Wu, Yunxiang Ge, Di Ma, Xue Pang, Yingyu Cao, Xiaofei Zhang, Yu Pan, Tong Zhang, Weibei Dou

**Affiliations:** ^1^School of Rehabilitation Medicine, China Rehabilitation Research Center, Capital Medical University, Beijing, China; ^2^Department of Rehabilitation Medicine, Beijing Tsinghua Changgung Hospital, School of Clinical Medicine, Tsinghua University, Beijing, China; ^3^Department of Electronic Engineering, Beijing National Research Center for Information Science and Technology (BNRist), Tsinghua University, Beijing, China; ^4^School of Mechanical Engineering, Beijing Institute of Petrochemical Technology, Beijing, China; ^5^Department of Clinical Epidemiology and Biostatistics, Beijing Tsinghua Changgung Hospital, Tsinghua University, Beijing, China

**Keywords:** motor-imagery brain-computer interface, regression analysis, stroke, upper limb, rehabilitation

## Abstract

**Objective:** Upper limb (UL) motor function recovery, especially distal function, is one of the main goals of stroke rehabilitation as this function is important to perform activities of daily living (ADL). The efficacy of the motor-imagery brain-computer interface (MI-BCI) has been demonstrated in patients with stroke. Most patients with stroke receive comprehensive rehabilitation, including MI-BCI and routine training. However, most aspects of MI-BCI training for patients with subacute stroke are based on routine training. Risk factors for inadequate distal UL functional recovery in these patients remain unclear; therefore, it is more realistic to explore the prognostic factors of this comprehensive treatment based on clinical practice. The present study aims to investigate the independent risk factors that might lead to inadequate distal UL functional recovery in patients with stroke after comprehensive rehabilitation including MI-BCI (CRIMI-BCI).

**Methods:** This prospective study recruited 82 patients with stroke who underwent CRIMI-BCI. Motor-imagery brain-computer interface training was performed for 60 min per day, 5 days per week for 4 weeks. The primary outcome was improvement of the wrist and hand dimensionality of Fugl-Meyer Assessment (δFMA-WH). According to the improvement score, the patients were classified into the efficient group (EG, δFMA-WH > 2) and the inefficient group (IG, δFMA-WH ≤ 2). Binary logistic regression was used to analyze clinical and demographic data, including aphasia, spasticity of the affected hand [assessed by Modified Ashworth Scale (MAS-H)], initial UL function, age, gender, time since stroke (TSS), lesion hemisphere, and lesion location.

**Results:** Seventy-three patients completed the study. After training, all patients showed significant improvement in FMA-UL (Z = 7.381, *p* = 0.000^**^), FMA-SE (Z = 7.336, *p* = 0.000^**^), and FMA-WH (Z = 6.568, *p* = 0.000^**^). There were 35 patients (47.9%) in the IG group and 38 patients (52.1%) in the EG group. Multivariate analysis revealed that presence of aphasia [odds ratio (OR) 4.617, 95% confidence interval (CI) 1.435–14.860; *p* < 0.05], initial FMA-UL score ≤ 30 (OR 5.158, 95% CI 1.150–23.132; *p* < 0.05), and MAS-H ≥ level I+ (OR 3.810, 95% CI 1.231–11.790; *p* < 0.05) were the risk factors for inadequate distal UL functional recovery in patients with stroke after CRIMI-BCI.

**Conclusion:** We concluded that CRIMI-BCI improved UL function in stroke patients with varying effectiveness. Inferior initial UL function, significant hand spasticity, and presence of aphasia were identified as independent risk factors for inadequate distal UL functional recovery in stroke patients after CRIMI-BCI.

## Introduction

Previous studies have reported that 85% of stroke survivors suffer from upper limb (UL) dysfunction, which has significant long-term effects on activities of daily living (ADL), leisure activities, and work (1). Upper limb motor function recovery, especially distal function, is one of the main goals of stroke rehabilitation. Only 12% of patients with stroke regain full UL function, while other patients require long-term care (2). Brain-computer interface (BCI) is an interactive system for internal and external environment that directly reflects brain activity (3). Motor-imagery brain-computer interface (MI-BCI) can quantify and reinforce feedback from motor imagery tasks and affect changes in neural network plasticity. For patients with stroke, MI-BCI training significantly improves UL motor function (4), electromyography signals (EEG) (5), joint mobility, daily living skills (6), mood (7), and brain network connectivity (8).

The effect of BCI is multidimensional, including the improvement of the clinical score and some subclinical indicators. Previous studies have shown significant improvement in UL function after MI-BCI training in patients with stroke (9, 10). Subclinical effects with improvement in the neuroelectrophysiological index have also been observed (11, 12). The mechanism of motor function recovery in the distal UL differs from that in the proximal UL. Most patients with stroke with inadequate overall UL functional recovery show predominantly improved proximal UL function, for instance, shoulder and elbow function, which results from non-decussated corticospinal fibers stemming from the unaffected hemisphere (13, 14). However, the distal UL function is markedly affected by the integrity of the corticospinal tract (CST) (12), which lacks a compensatory mechanism and therefore recovers inadequately. Motor function in the distal UL has a substantive effect on ADL (10, 15).

Most stroke patients receive comprehensive treatment that not only includes MI-BCI but also routine training. Multiple factors affect the prognosis of UL functional recovery. First, there is a large variation in user performance between healthy individuals, and the possible factors include cognitive function, sensory, motor imagery ability (16), psychological factors (17), age (18), gender (19), dominant hand (20), sensorimotor rhythm bias (21), cortical gray matter volume, and medical treatment. Second, studies on the prognosis prediction of UL motor recovery in patients with stroke have incorporated age, gender, dominant hand, lesion of hemisphere and location, initial UL function, and presence of comorbidities into models that can predict UL recovery type up to 6 months after onset (22).

In studies on MI-BCI fitness, gender, age, event-related potential, classification accuracy, neuropsychological score (23), EEG laterality index, and cortical activation intensity (24) were found to be useful to predict the performance of stroke patients who could manipulate MI-BCI devices, and the symmetry of the EEG signal could be used as a predictor of the degree of UL improvement (25, 26). Subacute phase is an essential rehabilitation period for patients with stroke, and an increasing number of studies have included subacute stroke patients in BCI studies. However, the effect of these factors on the effectiveness of comprehensive treatment including MI-BCI in patients with stroke is unclear. The elucidation of this aspect will enable us to better define the indications for MI-BCI, guide the development of individualized rehabilitation programs, and improve treatment effectiveness in patients with stroke.

Therefore, the present study aimed to investigate the independent risk factors that might lead to inadequate distal UL recovery of patients with stroke after comprehensive rehabilitation including MI-BCI (CRIMI-BCI).

## Subjects and Methods

### General Information

Eighty-two patients with ischemic stroke hospitalized at Tsinghua Changgung Hospital, Beijing, between January 2018 and December 2019 were recruited for the study. The diagnostic criteria were based on the “Consensus on Clinical Research Specifications for Acute Stroke in China 2018,” along with differential diagnosis by magnetic resonance imaging (MRI) or computerized tomography (CT) (27). The inclusion and exclusion criteria were chosen according to a previous study (8).

#### Inclusion Criteria

The following inclusion criteria were used: (1) age 18–75 years; (2) sufficient cognition [Montreal Cognitive Scale (MOCA) score >20]; (3) a history of first-ever unilateral brain lesion confirmed by MRI; (4) stroke occurrence [time since stroke (TSS)] 1–6 months prior to inclusion; (5) moderate to severe UL paralysis (Brunnstrom stages ≤ IV); and (6) right-handedness (Edinburgh Handedness Inventory score ≥40).

#### Exclusion Criteria

The following exclusion criteria were used: (1) severe spasticity of the affected hand [Modified Ashworth Scale (MAS) score ≥3]; (2) open wound or deformity of the affected UL; (3) visual field deficit or unilateral spatial neglect; (4) severe aphasia [Boston Diagnostic Aphasia Examination (BDAE) score <3]; (5) currently undergoing antipsychotic treatment; (6) severe dystonia and/or involuntary movements; (7) other severe neurological disorders such as epilepsy; and (8) currently undergoing neuromodulation treatment.

**Figure 1:**
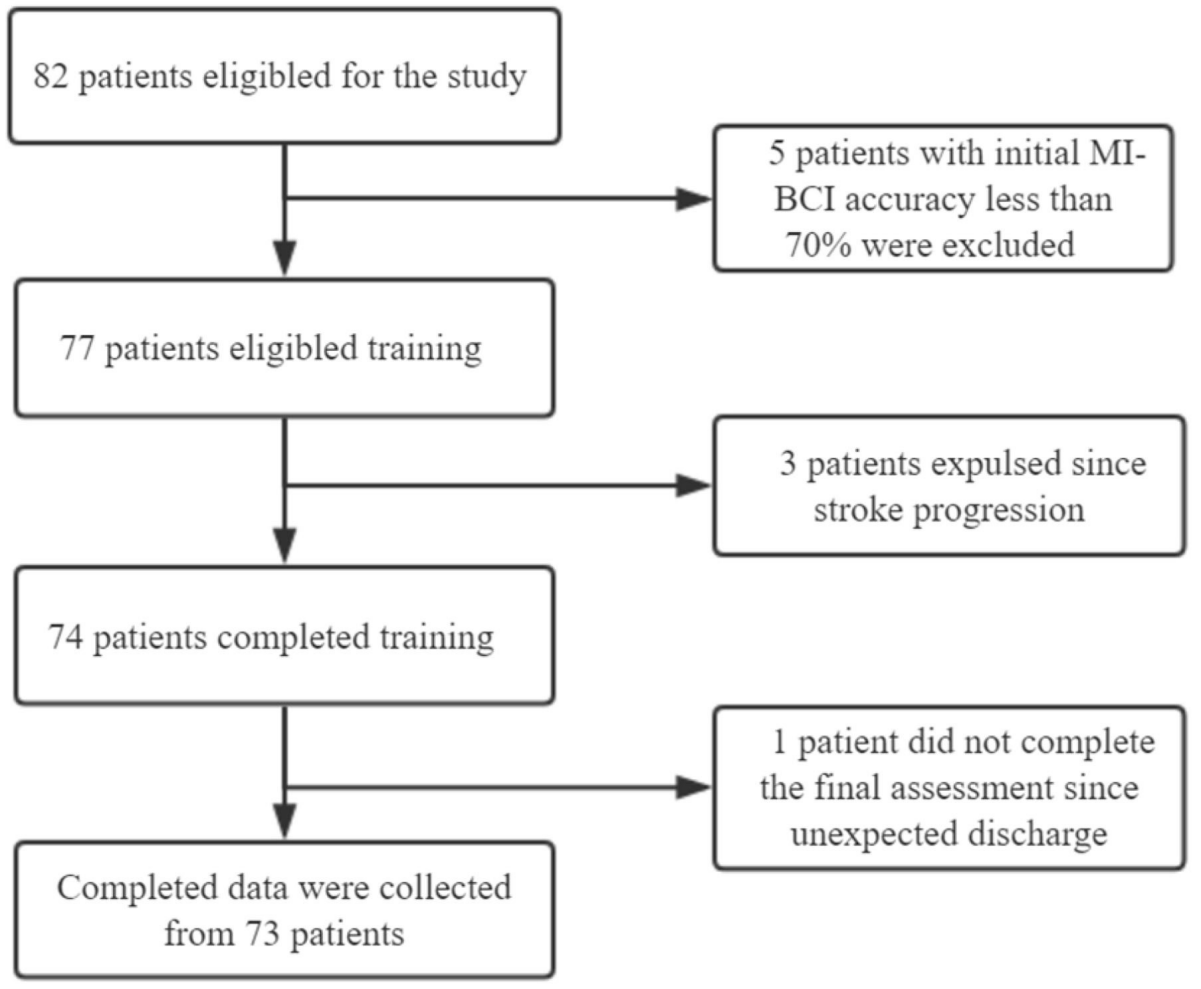
Experimental flow chart.

Nine patients were excluded at follow-ups conducted during the study due to changes in their conditions. Complete data were collected from 73 patients. The experimental flow chart is shown in Figure 1.

The study was approved by the Ethics Committee of Beijing Tsinghua Changgung Hospital (No. 18172-0-02) and registered at http://www.chictr.org.cn (No. ChiCTR1900022128). All patients signed an informed consent form prior to the trial. The following data were obtained from patients with stroke: gender, age, TSS, lesion hemisphere and location, and presence of concomitant aphasia. The lesion location was staged according to the Oxfordshire Community Stroke Study (OCPS) (28, 29). Aphasia diagnostic criteria were determined by the Aphasia Battery of Chinese (ABC), aphasia diagnostic criteria set as total score <25 (30).

General information of the 73 included patients is shown in Table 1.

**Table 1:** General information of 73 patients.

	**Mean/Median**	**Classification**	**Number**	***n* (%)**
Age (year)	61.00 (46.00, 67.00)	<65	50	68.49
		≥65	23	31.51
TSS (month)	3.00 (2.00, 5.00)	≤ 6	60	82.19
		>6	13	17.81
Lesion		TACI	29	39.73
		PACI	44	60.27
Gender		Female	25	34.25
		Male	48	65.75
Aphasia		With	49	67.12
		Without	24	32.88
Affected hemisphere		Right	31	42.47
		Left	42	57.53

*PACI, Partial anterior circulation infarct; TACI, Total anterior circulation infarct*.

### EEG Acquisition

Scalp EEG potentials were collected from eight dry electrodes (according to 10–20 International System), band-passed by 2–60 Hz filter and a notch filter (48–52 Hz) to remove artifacts and power line interference, respectively; digitized at 256 Hz, and amplified by a commercial EEG system (g. LADYbird, g. Tec Medical Engineering GmbH, Schiedlberg, Austria) and then processed by a computer.

EEG signals were grounded to a unilateral earlobe and referenced at the other one. Electrodes were positioned over FC3, FC4, C3, C4, CP3, CP4, C1, and C2. Signals from the C3 and C4 were used for device control. Motor function related electrodes were used for offline analyses (left hemisphere: FC3, C3, and CP3; right hemisphere: FC4, C4, and CP4). Mu suppression, which reflects Event-Related Desynchronization (ERD), was due to increased neural activity. The mu suppression score provides information about motor innervation, and was posted on the screen, encouraged patients to get higher scores. For mu suppression score computation, EEG data from C3 to C4 were transformed into the frequency domain by a Fourier transform algorithm with a Hanning window covering the EEG data during the video period of the paradigm. The equation of mu suppression scored are as follows (8):

$$\begin{array}{l} \text{Musupp}=-\frac{{\text{Mup}}_{\text{task}}-{\text{Mup}}_{\text{rest}}}{{\text{Mup}}_{\text{rest}\;}}\times 100 \end{array}$$

Musupp: Mu suppression score, Mup_test_: EEG power during MI; Mup_rest_: EEG power during the resting state.

### Comprehensive Rehabilitation Training Program

All patients received standard treatment for stroke in terms of medical care and rehabilitation, which consisted of conventional treatment, including an intensive occupational therapy focused on activities of daily live, such as grasping a toothpaste tube, eating, and reaching. A conventional treatment session lasted for 1 h per day, 5 days per week, for 4 weeks. MI-BCI training was applied based on conventional treatment. The detailed protocol refers to previous studies (8).

#### MI-BCI Training Paradigms

To facilitate MI performance, patients were given the opportunity to execute tasks with the affected and unaffected hand several times before MI-BCI training. Meanwhile they were instructed to perform only MI tasks and to avoid movement attempts of the affected UL.

MI-BCI training consisted of 20 sessions, five sessions per week, for 4 weeks. During each session, the patient was comfortably seated in a soundproofed room, with their affected hand resting in an exoskeleton hand. A video of the unaffected hand grasping/opening was presented on a screen in front of the patient to guide the MI task. The exoskeleton hand provided mechanical support and assistance to the affected hand based on the mu suppression algorithm calculated during the follow up trial.

1. Movement observation: a dark screen was first displayed for 2 s, followed by a white cross for 2 s. A vocal cue of “hand grasp” or “hand open” was displayed for 2 s. Then a video clip was displayed for a duration of 6 s. Patients were requested to observe the video and avoid blinking, coughing, chewing, and performing head movements.
2. Exoskeleton hand assistance: If the mu suppression score was above 20, the exoskeleton hand would assist the hand grasping/opening task during the following 3 s.
3. End of process: the mu suppression score was then shown for 2 s. The trial ended with the display of a dark screen for 2 s. During each session, the trial was repeated 100 times for one session, and video clips of the grasping and opening hand were shown randomly. Patients were permitted to rest for 1 min after 10 trials.

### Functional Evaluation

Before and after 20 training sessions, the motor function of the affected UL and muscle tone of the affected finger flexor were evaluated. A simplified version of the Fugl-Meyer Assessment UL (FMA-UL), which was without sensory, passive joint mobility, and pain and reflex sections, was used to assess the motor function of the affected UL (31). The total FMA-UL score was 60, which was obtained by summing the five items on the FMA-UL scale: shoulder, elbow, wrist, hand, and coordination/speed. The sum of the wrist and hand score (FMA-WH) was 24. The sum of the shoulder and elbow score (FMA-SE) was 30. A higher score for each dimension implied that the patient was more functional. The simplified FMA-UL score was categorized as severe (0–12), severe-moderate (13–30), moderate-mild (31–47), and mild (48–60) (2). Because the initial FMA-UL scores of most patients were between severe-moderate to moderate-mild, a score of 30 was selected as the watershed in the following regression analysis.

The MAS was used to measure the muscle tone of the affected finger flexors and recorded as MAS-H (32, 33). For statistical convenience, the scores were recorded as follows: level 0 was set as 1, level I–I+ as 2, level II as 3, and level III–IV as 4. Because the initial MAS-H scores of most patients were between 2 and 3, level I+ was selected as the watershed.

All assessments were performed by one trained therapist, and the assessor was unaware of the therapeutic condition of the patients. Functional evaluation of the 73 patients is shown in Table 2.

**Table 2:** UL function in 73 patients before and after training.

	**Pre**	**Post**	**Z**	***p***
FMA-UL	18 (8, 27.5)	30 (16, 45.5)	7.381	0.000^**^
FMA-SE	13 (8–19)	20 (12, 28.5)	7.336	0.000^**^
FMA-WH	3 (0, 8)	9 (4–16)	6.568	0.000^**^

** *p < 0.01*.

### Comparison of Group Characteristics

The 73 patients who completed all training and testing procedures were divided into two groups according to whether the increase in the FMA-WH score (δFMA-WH) was higher than two points before and after treatment. The differences between the two groups were compared in terms of gender, age, TSS, lesion hemisphere and location, presence of aphasia, MAS-H, and FMA-UL and its component score.

### Statistical Analysis

Data were analyzed using SPSS 22.0 statistical software, and measured values were expressed as mean and standard deviation (x ± s) or median and interquartile range (25th percentile, 75th percentile). Data were verified for normality of distributions with the Kolmogorov-Smirnov test (*n* > 50) or Shapiro-Wilk test (*n* < 50). Because of the ordinal nature of the data, the study data were compared between groups using independent sample *t*-test and Mann-Whitney U test. Data were compared within the group using an independent *t*-test and a paired-sample Wilcoxon test. Categorical variables were expressed as percentages and analyzed with the chi-square test or Fisher's exact test, and significance was set at *p* < 0.05. For regression analysis, the δFMA-WH score was used as the criterion for grouping. Binary logistic regression was performed on the basis of the results of univariate analysis for *p* < 0.10 and by incorporating the parameters into the model through the Forward LR method. The results of multivariate analysis were expressed as odds ratio (OR) and 95% confidence interval (CI). A two-sided *p* < 0.05 was considered to be statistically significant.

## Results

### Overall Efficacy of Comprehensive Rehabilitation

**Figure 2:**
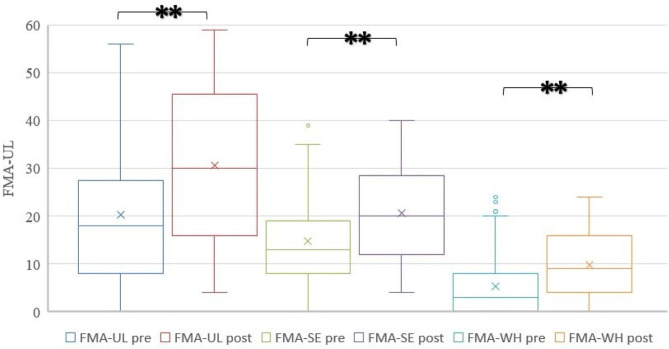
Improvement of 73 patients after treatment.

After comprehensive rehabilitation, 73 patients showed significant improvement in FMA-UL, FMA-SE, and FMA-WH (Wilcoxon signed-rank test, *p* < 0.000), as shown in Table 2 and Figure 2.

### Differences in Functional Recovery

It was difficult to perceive changes in hand function when δFMA-WH ≤ 2 points (33) and by referring to the definition of minimum clinically important difference (MCID). Hence, δFMA-WH ≤ 2 points before and after training was selected as the criterion for judging the efficacy (34, 35). The 73 patients were divided into the efficacy group (EG, with δFMA-WH > 2 points, *n* = 38, 52.1%) and the inefficacy group (IG, δFMA-WH ≤ 2 points, *n* = 35, 47.9%).

The difference in UL motor function status was compared between the two groups. The results showed no significant differences in the FMA-UL pre (Mann-Whitney U test, *p* = 0.584), FMA-SE pre (Mann-Whitney U test, *p* = 0.615), and FMA-WH pre (Mann-Whitney U test, *p* = 0.950) between the groups before training.

After training, the total score and the WH score in both EG and IG groups were significantly higher than those before the training [FMA-UL post (Mann-Whitney U test, *p* = 0.026) and FMA-WH post (Mann-Whitney U test, *p* = 0.001)]. The improvement in the total, SE, and WH scores of FMA-UL in the EG group was also significantly higher than that in the IG group [δFMA-UL (Mann-Whitney U test, *p* = 0.000), δFMA-WH (Mann-Whitney U test, *p* = 0.000), and δFMA-SE (independent samples *t*-test, *p* = 0.000)], as listed in Table 3 and Figure 3.

**Table 3:** Comparison of changes before and after training between groups.

	**EG (*n* = 38)**	**IG (*n* = 35)**	***t*/*Z***	***p***
FMA-UL pre	18,000 (12.0, 24.0)	12,000 (6.0, 40.0)	−0.548	0.584
FMA-SE pre	13,000 (10.0, 18.0)	10,000 (6.0, 24.0)	−0.503	0.615
FMA-WH pre	4,000 (0.0, 7.0)	2,000 (0.0, 13.0)	−0.063	0.950
FMA-UL post	32,000 (23.0, 47.3)	18,000 (8.0, 44.0)	−2.226	0.026^*^
FMA-SE post	21,000 (16.0, 28.3)	17,000 (8.0, 29.0)	−1.261	0.207
FMA-WH post	12,000 (7.8, 16.3)	4,000 (0.0, 13.0)	−3.406	0.001^**^
δFMA-UL	14,000 (9.8, 18.3)	4,000 (3.0, 5.0)	−6.264	0.000^**^
δFMA-SE	17.42 ± 7.06	13.09 ± 6.24	2.770^#^	0.007^**^
δFMA-WH	7,000 (4.0, 11.0)	1,000 (0.0, 2.0)	−7.388	0.000^**^

* *p < 0.05*,

** *p < 0.01*,

# *Mann-Whitney U test*.

**Figure 3:**
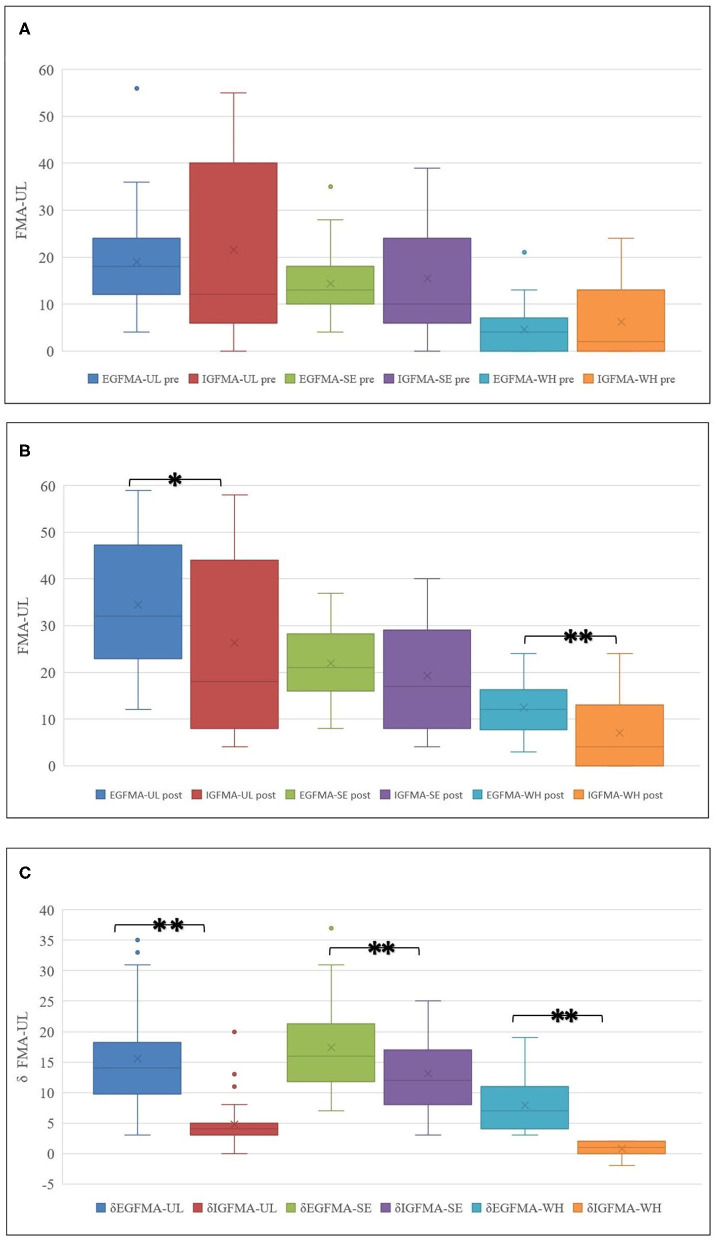
**(A–C)** UL function improvement in groups. Although the overall FMA-UL score improved, the FMA-WH scores in two patients regressed after training in IG. *p < 0.05, **p < 0.01.

### Risk Factor of Unfavorable Recovery

**Figure 4:**
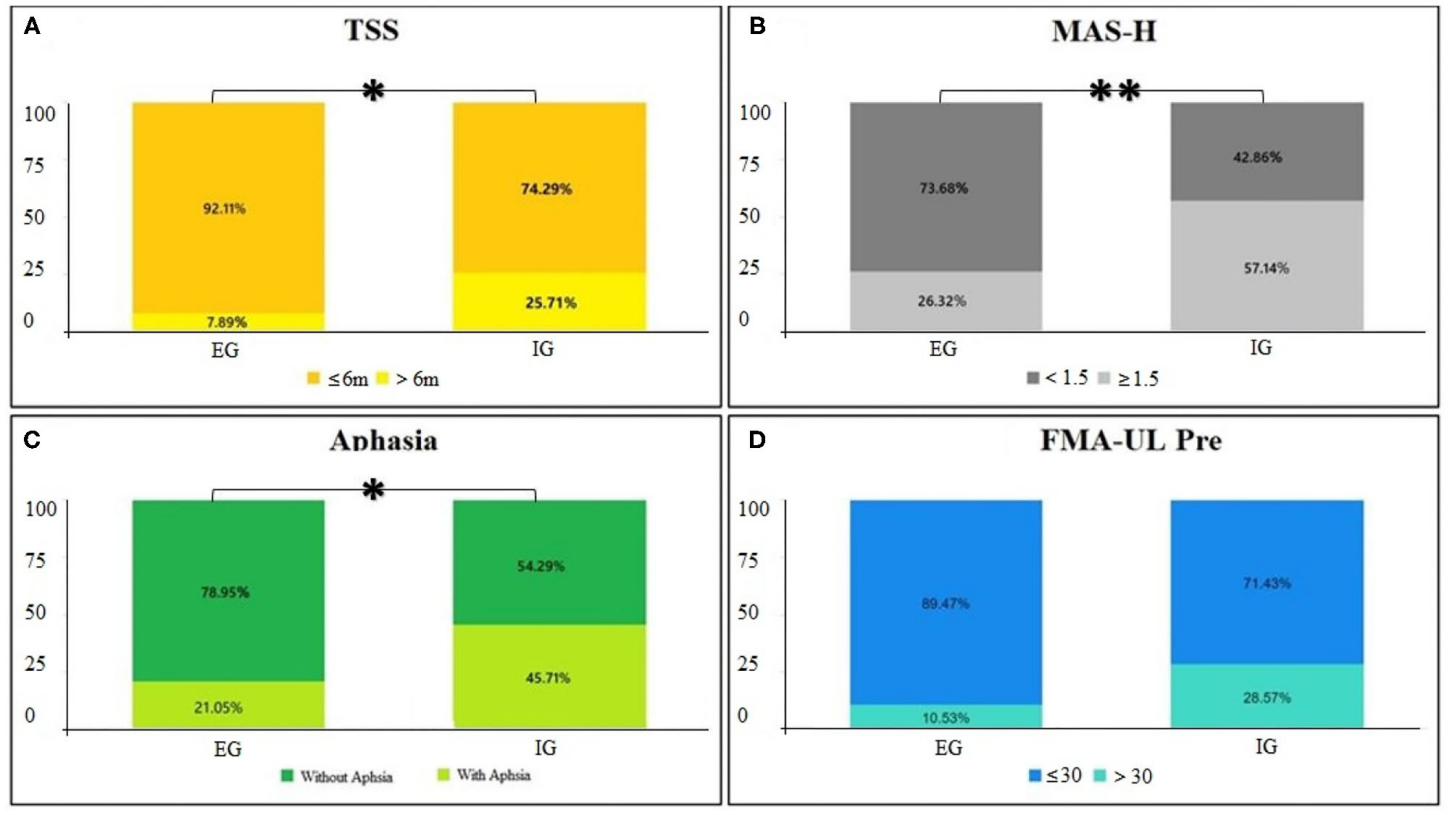
**(A–D)** Univariate analysis of characteristics. Cross graph of groups and classification for *p* < 0.05, **p < 0.01.

The univariate analysis revealed that TSS (chi-square test, *p* = 0.040), MAS-H (chi-square test, *p* = 0.007), presence of aphasia (chi-square test, *p* = 0.025), and FMA-UL pre (chi-square test, *p* = 0.05) might be associated with unfavorable hand function recovery after BCI training in patients with stroke. Age (chi-square test, *p* = 0.989), lesion (chi-square test, *p* = 0.205), gender (chi-square test, *p* = 0.626), and affected hemiplegia (chi-square test, *p* = 0.377) were not found to be relevant for the functional change of the distal UL after training in patients, as listed in Table 4 and Figure 4.

**Table 4:** Comparison of the characteristics between groups.

	**Classification**	**EG (%)**	**IG (*n* %)**	**Subtotal (*n* %)**	*****χ^2^*****	***p***
Age (year)	≤ 65	26 (68.42)	24 (68.57)	50 (68.49)	0	0.989
	>65	12 (31.58)	11 (31.43)	23 (31.51)		
TSS (month)	≤ 6	35 (92.11)	26 (74.29)	61 (83.56)	4.212	0.040^*^
	>6	3 (7.89)	9 (25.71)	12 (16.44)		
MAS-H	≤ 1+	28 (73.68)	15 (42.86)	43 (58.90)	7.152	0.007^**^
	>1+	10 (26.32)	20 (57.14)	30 (41.10)		
Lesion	TACI	15 (39.47)	19 (54.29)	34 (46.58)	1.606	0.205
	PACI	23 (60.53)	16 (45.71)	39 (53.42)		
Gender	Female	14 (36.84)	11 (31.43)	25 (34.25)	0.237	0.626
	Male	24 (63.16)	24 (68.57)	48 (65.75)		
Aphasia	Without	30 (78.95)	19 (54.29)	49 (67.12)	5.021	0.025^*^
	With	8 (21.05)	16 (45.71)	24 (32.88)		
Affected hemisphere	Left	18 (47.37)	13 (37.14)	31 (42.47)	0.780	0.377
	Right	20 (52.63)	22 (62.86)	42 (57.53)		
FMA-UL pre	≤ 30	34 (89.47)	25 (71.43)	59 (80.82)	3.827	0.050
	>30	4 (10.53)	10 (28.57)	14 (19.18)		

* *p < 0.05*,

** *p < 0.01*.

Binary logistic regression analysis was performed based on the results of univariate analysis for *p* < 0.10. Presence of aphasia [odds ratio (OR) 4.617, 95% confidence interval (CI) 1.435–14.860; *p* = 0.010], FMA-UL pre ≤ 30 (OR 5.158, 95% CI 1.150–23.132; *p* < 0.032), and MAS-H ≥ level I+ (OR 3.810, 95% CI 1.231–11.790; *p* = 0.020) were found to be risk factors for inadequate distal UL recovery in patients with stroke after CRIMI-BCI training, as shown in Table 5.

**Table 5:** Binary logistic regression of risk factors.

	***B***	***SE***	***Z*-value**	**Wald χ^2^**	***P***	**OR**	**OR (95%CI)**
MAS-H	1.338	0.576	2.321	5.386	0.020^*^	3.810	1.231–11.790
FMA-UL	1.641	0.766	2.143	4.591	0.032^*^	5.158	1.150–23.132
TSS	0.515	0.812	0.635	0.403	0.526	1.674	0.341–8.222
Aphasia	1.530	0.596	2.565	6.579	0.010^*^	4.617	1.435–14.860

*B, estimate coefficient; SE, standard error; OR, odds ratio; CI, confidence interval*.

* *p < 0.05*.

## Discussion

The results of the present study showed that for the target population, CRIMI-BCI significantly improved the overall and distal motor function of the UL in stroke patients, but the degree of improvement varied. Classification according to distal functional improvement showed that although no significant differences were observed in UL motor function before training, the proportion of patients with aphasia, TSS > 6 months, and finger flexor tone >1+ grade on the affected hand was significantly higher in the IG group. Regression analysis showed that significant hand spasticity, poor initial function, and concomitant aphasia were the important factors that influenced the inadequate distal UL motor function after CRIMI-BCI in this study.

The results of the present study showed that CRIMI-BCI improved UL function, including distal motor function, in patients with stroke. This result is consistent with many studies (6, 25, 35). Bundy DT found significant improvements in grip strength, grasp, and pinch function in stroke patients with chronic moderate to severe UL paresis, after using BCI combined with exoskeleton training in the unaffected hemisphere (35). Frolov et al. showed that in patients with stroke, BCI combined with exoskeleton training showed a significant advantage in the grip and pinch function of the affected finger as compared to exoskeleton training alone (25).

Previous studies on factors influencing the clinical outcome of MI-BCI have shown no correlation between spasticity and training outcome in patients with stroke. However, the effect of spasticity on UL motor function was demonstrated by numerous studies on the prediction of motor function in stroke patients. The present study showed that the MAS-H score was an independent factor that influenced inadequate functional improvement of the distal UL in patients after CRIMI-BCI. Finger and wrist flexor spasticity is very common in patients with stroke, and the recovery of wrist and hand movements vary greatly among patients with different degrees of spasticity. Patients with severe spasticity have minimal improvement in the FMA of the hand (34, 36). In contrast, patients with moderate and mild spasticity exhibit greater hand motion recovery. Hand spasticity affects the recovery of random finger movements as well as grasp and release functions. Spasticity can also impede motor learning ability after stroke (37). The severity of distal spasticity directly reflects the severity of the CST injury, as there is no compensation of the ipsilateral motor conduction. Spasticity of the hand is an extremely critical influencing factor in the recovery of motor function after stroke (36). The understanding of the relationship between hand spasticity and the effect of MI-BCI training in patients with stroke can be helpful in designing individualized rehabilitation programs.

Previous studies have suggested that complications such as depression, cognitive impairment, aphasia, hemianopia, and unilateral neglect after stroke also affect the recovery of motor function (38). The presence of spontaneous speech is a favorable factor for the recovery of motor function (39, 40). The present study showed that concomitant aphasia was an independent influencing factor of modest functional improvement of the distal UL in the patients. A possible reason for this was that although patients with concomitant aphasia in this study had mild speech impairment, the training process involved more complex instructions such as hearing vocal signals—watching videos—closing eyes—completing motor imagery—opening eyes to prepare for the next cycle; hence, speech dysfunction not only affected the reception and expression of information by the patients, but it may also have involved factors such as attention and working memory, resulting in inefficiency training and inadequate rehabilitation. Further research is necessary to understand the effect of aphasia on the effectiveness of MI-BCI in terms of the type, severity, and mechanism of the disorder.

Many studies have confirmed that the performance of MI-BCI manipulation does not correlate with motor function. However, in most stroke prediction models, the initial UL function score significantly affects prognosis. The patients in the present study had no significant differences in FMA-UL pre as observed in Mann-Whitney U test; all of these patients had moderate or severe impairment. However, the difference in data dispersion between the groups can be observed from the box plot. There was an inter group difference in the trend of the initial FMA-UL degree (classified by 30 points), and a further increase in sample size might have cleared the difference. We incorporated the FMA-UL pre-score into the regression analysis based on clinical experience and prognostic studies. However, the initial motor function still showed a significant effect on treatment outcome in the regression analysis; this finding is consistent with the general pattern of UL functional recovery in patients with stroke. The result may be related to the initial FMA-UL score distribution or the interaction between the variables.

It is worth noting that, unlike previous studies, the present study did not show any influence of TSS, age, gender, and lesion on the effect of CRIMI-BCI. This may be explained by the distinctive population scope of the study. For example, in the intragroup comparison, the number of cases with TSS > 6 months was significantly greater in the IG group than in the EG group. However, in the multivariate analysis, TSS did not constitute an independent risk factor affecting the unfavorable functional recovery of the distal UL in stroke patients. Age has been reported to influence the performance of MI-BCI in healthy individuals, with older patients showing reduced EEG power, laterality, and significantly lower discrimination accuracy than younger patients (40, 41). Advanced age is also an influential factor in the inadequate prognosis of UL function after stroke (22, 42, 43). Regarding the effect of gender on outcome, many studies on the prognosis of stroke have shown that male patients have a better prognosis than female patients. However, Randolph reported that male patients had poorer ability to regulate μ-rhythms in the EEG and poorer control of the MI-BCI system than female patients (19). In terms of the location of lesions, the anterior putamen, internal capsule, thalamus, periventricular white matter, and premotor cortex were associated with inadequate UL recovery in patients with hemorrhagic stroke (44). Studies on chronic ischemic stroke have shown poorer recovery in patients with internal capsule injury (45). The prognosis of the TACI was worse than that of the other types (46, 47). In the present study, most patients with complete anterior circulation and severe posterior circulation could not complete MI-BCI training because of other comorbidities. In contrast, there is a POCI type in OCPS, but no patient with this type was not recruited in this study. This might be because such types of patients generally face less obstacles and therefore hardly meet the test conditions. This may be related to the generally mild motor dysfunction of these patients. Therefore, the results of the present study were limited to the current population for whom MI-BCI is indicated, and the duration of the disease was restricted to the subacute phase. Additionally, cognitive impairment, severe aphasia, severe spasticity, depression, hemianopia, unilateral neglect, and other comorbidities were excluded, and the effect of the dominant hand was limited. The patient population in the present study was more homogeneous than that observed in actual clinical patients, and no remarkable differences were observed in TSS, age, gender, and lesion. Hence, the effect of the training might not be evident. This suggested that the study of factors influencing the effect of CRIMI-BCI on patients with stroke was very complicated, and the findings need to be tailored to its scope of application. Moreover, there is an urgent need to combine neuroimaging and neurophysiological investigations in the next step of this study.

The results of the present study answered the initial queries and showed the following findings: (1) CRIMI-BCI can improve UL motor function, especially the distal function, in patients with stroke in the subacute stage; (2) a heterogeneity was observed in the effect of CRIMI-BCI on distal UL function, with significantly better overall and distal UL function improvement in EG patients than in IG patients; and (3) in stroke patients with subacute phase UL paralysis who underwent CRIMI-BCI, poor initial UL function, severe hand spasticity, and concomitant aphasia were the influencing factors of inadequate distal function recovery.

## Limitations

The generalization of these results is subject to certain limitations. First, although previous studies have compared the effects of conventional therapy with BCI training on patients with subacute stroke, the present study did not include a control group, and therefore, it cannot be determined whether the abovementioned findings differ from those of patients with conventional stroke. Second, the sample size was insufficient and limited by the strict screening requirement of the patients and the long-term training duration. Moreover, there was inadequate inclusion of factors influencing clinical outcomes, including NIHSS score and some early clinical predictors such as the presence of shoulder shrugging, active finger extension, and grip strength of the affected hand. Third, indicators reflecting the ability to manipulate MI-BCI, including classification rate and EEG activity, were lacking. Fourth, neuroimaging-related evidence was lacking. In addition, long-term and interaction effects were not analyzed. Therefore, controlled, long-term studies with larger sample siues, more factors, and neuroimaging and neurophysiological evidence are needed to further validate the results of the present study.

## Conclusion

The present study aimed to investigate the factors influencing the effect of CRIMI-BCI on the functional recovery of the distal UL in patients with stroke. The main results suggest that CRIMI-BCI improved overall and distal UL function in patients with subacute stroke, but that the outcomes were varied. Poor initial UL function, severe hand spasticity, and concomitant aphasia were independent risk factors for inadequate MI-BCI training outcomes. This study was restricted to a specific population with subacute stroke who underwent CRIMI-BCI. It clarified the effect of BCI on distal UL function and explored further indications, prognosis, and protocols for rehabilitation. The study findings can be applied to design individualized rehabilitation programs for patients with stroke.

## Data Availability Statement

The datasets presented in this study can be found in online repositories. The names of the repository and accession number can be found below: Chinese Clinical Trial Registry (ChiCTR), http://www.chictr.org.cn, No. ChiCTR1900022128.

## Ethics Statement

The studies involving human participants were reviewed and approved by Ethics Committee of Beijing Tsinghua Changgung Hospital. The patients/participants provided their written informed consent to participate in this study.

## Author Contributions

QW, YP, WD, and TZ designed the study. DM, XP, and YC performed experiments. QW and YG wrote the paper. All authors contributed to the article and approved the submitted version.

## Conflict of Interest

The authors declare that the research was conducted in the absence of any commercial or financial relationships that could be construed as a potential conflict of interest.
